# Probing Physical Oxidation State by Resonant X‐ray Emission Spectroscopy: Applications to Iron Model Complexes and Nitrogenase

**DOI:** 10.1002/anie.202015669

**Published:** 2021-03-23

**Authors:** Rebeca G. Castillo, Anselm W. Hahn, Benjamin E. Van Kuiken, Justin T. Henthorn, Jeremy McGale, Serena DeBeer

**Affiliations:** ^1^ Department of Inorganic Spectroscopy Max Planck Institute for Chemical Energy Conversion Stiftstrasse 34–36 45470 Mülheim an der Ruhr Germany; ^2^ European XFEL Holzkoppel 4 22869 Schenefeld Germany

**Keywords:** iron complexes, molybdenum, nitrogenase, oxidation states, valence-to-core X-ray emission spectroscopy

## Abstract

The ability of resonant X‐ray emission spectroscopy (XES) to recover physical oxidation state information, which may often be ambiguous in conventional X‐ray spectroscopy, is demonstrated. By combining Kβ XES with resonant excitation in the XAS pre‐edge region, resonant Kβ XES (or 1s3p RXES) data are obtained, which probe the 3d^*n*+1^ final‐state configuration. Comparison of the non‐resonant and resonant XES for a series of high‐spin ferrous and ferric complexes shows that oxidation state assignments that were previously unclear are now easily made. The present study spans iron tetrachlorides, iron sulfur clusters, and the MoFe protein of nitrogenase. While 1s3p RXES studies have previously been reported, to our knowledge, 1s3p RXES has not been previously utilized to resolve questions of metal valency in highly covalent systems. As such, the approach presented herein provides chemists with means to more rigorously and quantitatively address challenging electronic‐structure questions.

## Graphical abstract

Assigning transition‐metal physical oxidation states is a major goal of X‐ray spectroscopy. However, competing influences of covalency and d‐count on Kβ XES spectra often make assignments ambiguous. It is now shown that resonant Kβ X‐ray emission spectroscopy (RXES) yields unambiguous oxidation‐state determinations for iron monomers, dimers, cubanes, and metalloenzymes.

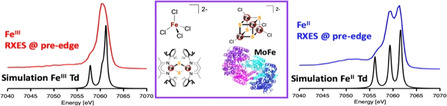

## Introduction

The concept of oxidation state is fundamental to how chemists communicate about electronic structure and chemical reactivity. We distinguish formal oxidation states, which are effectively used for bookkeeping of electrons, from physical oxidation states, which are assigned based on experimental observables. Spectroscopic methods play a key role in the identification of physical oxidation states. X‐ray spectroscopy is frequently utilized to assign oxidation states in transition metal (TM) complexes, largely due to its element selectivity and involvement of core electron excitations which provide an experimental means to probe the changes in effective nuclear charge at the selected photoabsorbing atom. Metal K‐edge X‐ray absorption spectroscopy (XAS) serves as a reasonable fingerprint for changes in metal oxidation state, with the 1s to 4p edge features of first‐row transition metals (TMs) shifting up by ∼1 eV per unit change in oxidation state.[^1^, ^2^, ^3^, ^4^, ^5^, ^6^, ^7^, ^8^, ^9^, ^10^, ^11^] However, as other factors, including ligand identity, coordination number, and metal spin state, contribute to the rising edge position,[^10^, ^12^, ^13^, ^14^, ^15^, ^16^, ^17^] caution must be exercised in using the metal K‐edge energies as a generalized measure of oxidation state. This observation has led to significant debate in the literature as to how edges can be quantitatively interpreted.[^18^, ^19^, ^20^]

Metal L‐edge XAS (2p→3d)[^21^, ^22^, ^23^, ^24^, ^25^, ^26^] can also provide covalency and metal oxidation state information, but experimental intensity and covalency can only be correlated through computational studies. These correlations may be further biased by the computational protocol or individual interpretation.[^19^, ^27^] In this regard, very similar Cu L‐edge data of formal Cu^III^ complexes have been used to both support^[27]^ and dismiss^[19]^ a Cu^III^ physical oxidation state assignment.

In addition to XAS, one can also utilize X‐ray emission spectroscopy (XES) to obtain insight into the physical oxidation state and spin state of a TM absorber.[^28^, ^29^, ^30^, ^31^, ^32^, ^33^, ^34^, ^35^, ^36^, ^37^, ^38^, ^39^] The Kβ mainlines in first‐row TMs correspond to the emission process that occurs when an electron in the 3p shell refills the 1s core‐hole created upon ionization, resulting in a 3d^*n*^3p^5^ final state (FS). Hence, Kβ mainlines are dominated by 3p–3d exchange coupling that splits the mainline into Kβ_1,3_ and Kβ′ features and provides information about the number of unpaired d‐electrons (where a larger number of unpaired d‐electrons results in larger 3p–3d exchange coupling).[^36^, ^39^, ^40^, ^41^] However, due to the covalent dilution of 3d character by ligand‐based orbitals, a decrease in d‐count due to oxidation may be countered by the corresponding increase in metal–ligand covalency, yielding a similar net 3p–3d exchange splitting for molecules of different d‐counts.[^14^, ^42^]

Consequently, it appears that there are many ways for X‐ray spectroscopic measurements to lead to ambiguous conclusions. In our view, this issue is best addressed by taking a holistic view of all available spectroscopic data (both X‐ray based and other methods) to arrive at a consistent picture of the electronic structure. In a recent study^[14]^ of dimeric [Fe_2_S_2_]^2+/1+/0^ complexes spanning three oxidation states, we showed that the rising edges of the diferrous and mixed valent iron dimers are nearly superimposable and that the Kβ XES mainlines of the full [Fe_2_S_2_]^2+/1+/0^ series are essentially identical. Despite this, crystallographic data, Mössbauer, and calculations of spectroscopic data supported diferrous, mixed‐valent, and diferric oxidation states assignments for these complexes, respectively. By combining spectroscopic results with computation, we were able to highlight the relative strengths and weaknesses of each spectroscopic approach in assessing the oxidation state.

This however, raises an important question: is there a spectroscopic approach that allows our concepts of metal valency, covalency, and oxidation state to be more rigorously tested and experimentally assessed? It is here that we draw inspiration from the words of Linus Pauling in his 1948 Liversidge lecture where he stated with regard to our understanding of valency, “…and we may hope that powerful methods of investigation that are not yet known will be discovered”.^[43]^ In our view, resonant X‐ray emission spectroscopy (RXES also known as resonant inelastic X‐ray scattering (RIXS)) is a tool with the potential to fulfill this role. RXES has historically been a tool for physicists,[^44^, ^45^, ^46^, ^47^, ^48^] and, while various types of RXES experiments have recently been utilized by the chemistry community,[^49^, ^50^, ^51^, ^52^, ^53^, ^54^, ^55^, ^56^, ^57^] it is clear that we are still evolving our understanding of the rich chemical information content of these spectra. In particular, significant progress has been made utilizing 2p3d and 1s2p RXES to extract d–d transition energies and L‐edge‐like information, respectively.[^49^, ^52^, ^53^, ^58^, ^59^] Additionally, 1s3p RXES has been utilized to obtain spin and oxidation state selective XAS, often referred to as Kβ‐detected XAS.[^60^, ^61^, ^62^, ^63^, ^64^] However, to our knowledge the detailed chemical and electronic structural information contained in the 1s3p RXES spectra themselves has yet to be fully explored.

**Figure 1:**
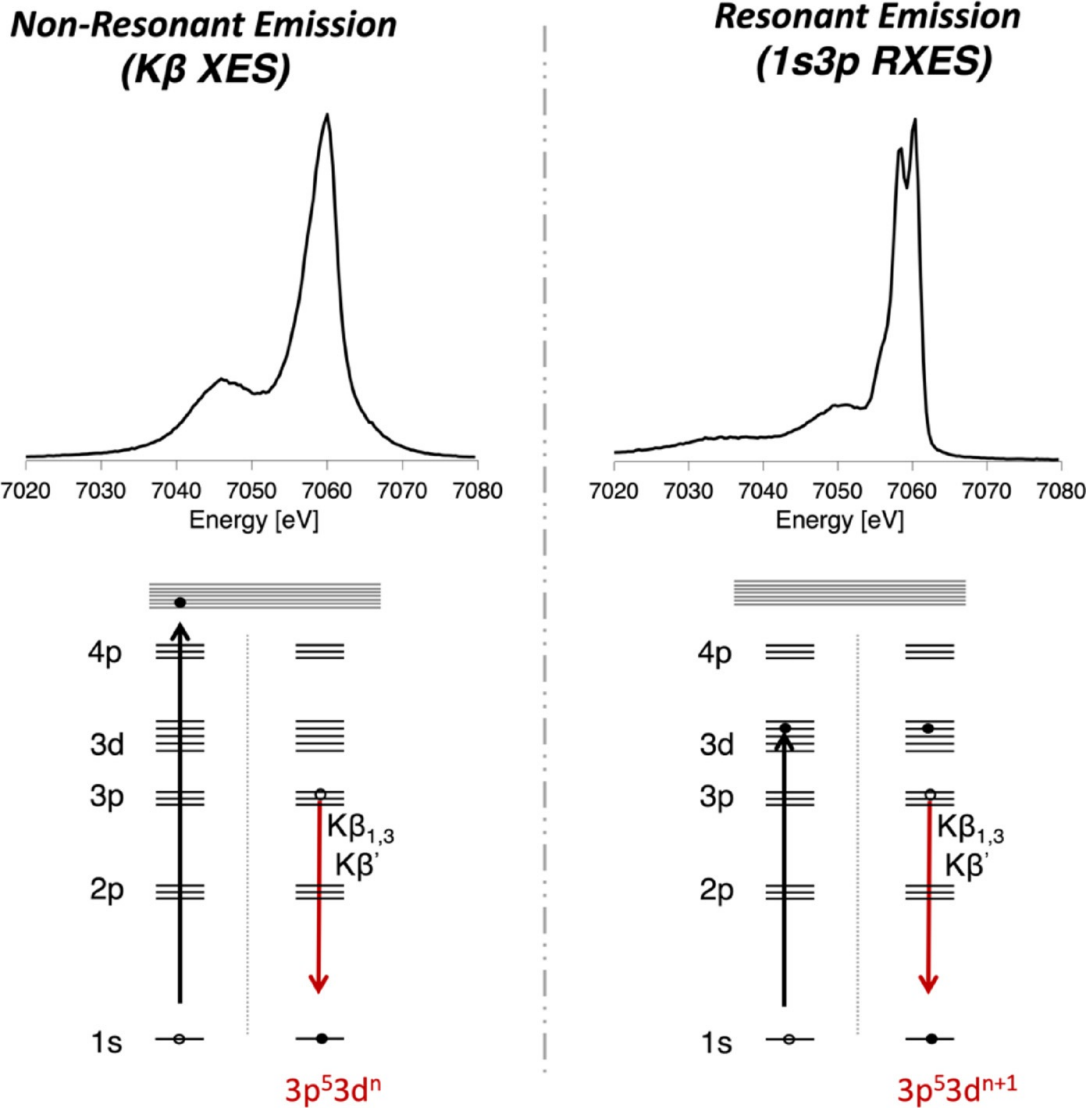
Comparison of the Kβ XES (top left) and 1s3p RXES spectra (top right) for [Fe^II^Cl_4_]^2−^, with the corresponding excitation and decay processes shown below each spectrum.

**Figure 2:**
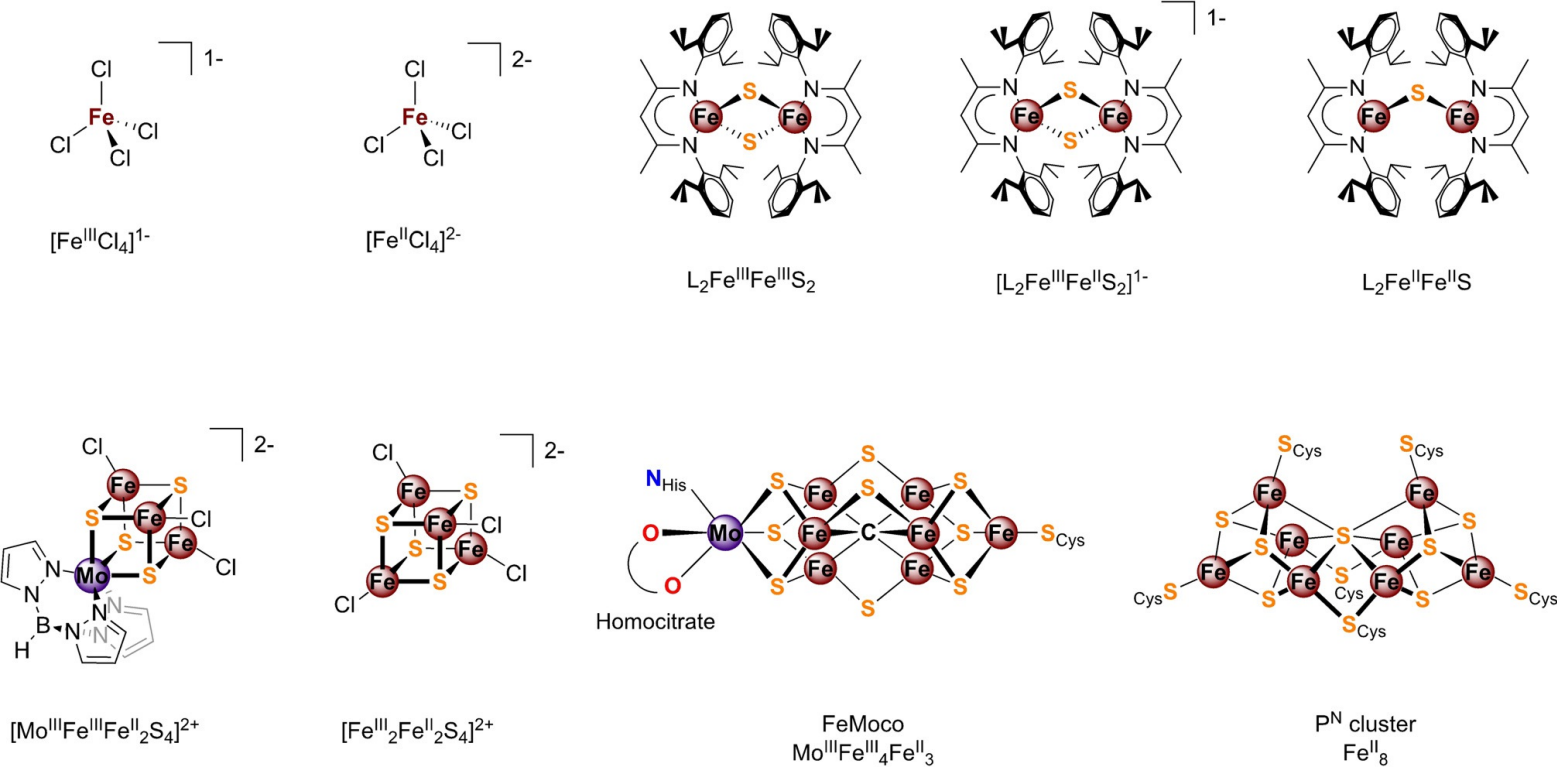
Complexes investigated in this study. Top: [Fe_4_S_4_Cl_4_]^1−^ and[Fe_4_S_4_Cl_4_]^2−^, the β‐diketiminate‐supported (L^1−^) iron sulfur complexes L_2_Fe^III^Fe^III^S_2_, [L_2_Fe^III^Fe^II^S_2_]^1−^, and L_2_Fe^II^Fe^II^S. Bottom: FeMoco cofactor and P‐cluster, contained in MoFe protein, [Mo^III^Fe^III^Fe^II^2S_2_]^2+^ ([MoFe_3_S_4_]^2+^), and [Fe^III^_2_Fe^II^2S_2_]^2+^ ([Fe_4_S_4_]^2+^).

Below, we present 1s3p RXES (Kβ RXES) as a means to recover physical oxidation state information, which may be lost in classical non‐resonant emission (Kβ XES) measurements due to the countering effects of covalency and metal d‐count (Figure 1). A systematic series, including ferrous and ferric tetrachlorides, as well as iron sulfur dimers, tetranuclear clusters, and the MoFe protein of nitrogenase (Figure 2), was studied by non‐resonant and resonant Kβ XES, as well as Kβ‐detected high energy resolution fluorescence detected (Kβ HERFD) XAS, which allows for higher resolution than standard XAS.[^5^, ^9^]

In a Kβ RXES experiment, the 1s electron is excited into specific unoccupied orbitals by tuning the incident excitation energy. For instance, when resonantly exciting into the pre‐edge region of the XAS spectra (dominated by 1s to 3d transitions), 1s3p RXES spectra are obtained, with 3p^5^3d^*n*+1^ FS. By performing both resonant and non‐resonant XES, we are able to experimentally differentiate between different 3d^*n*^ counts despite the changes in covalency. As such, the presented 1s3p RXES approach provides chemists with a tool for more rigorously assigning physical oxidation states. To our knowledge the ability to use 1s3p RXES to assess oxidation states in highly covalent systems has not previously been reported. The extension of 1s3p RXES to the MoFe protein of nitrogenase further establishes the viability of this method for studying electronic structural questions in biological systems.

## Results and Discussion

### Fe K‐Edge XAS and (Non‐resonant) Kβ XES

**Figure 3:**
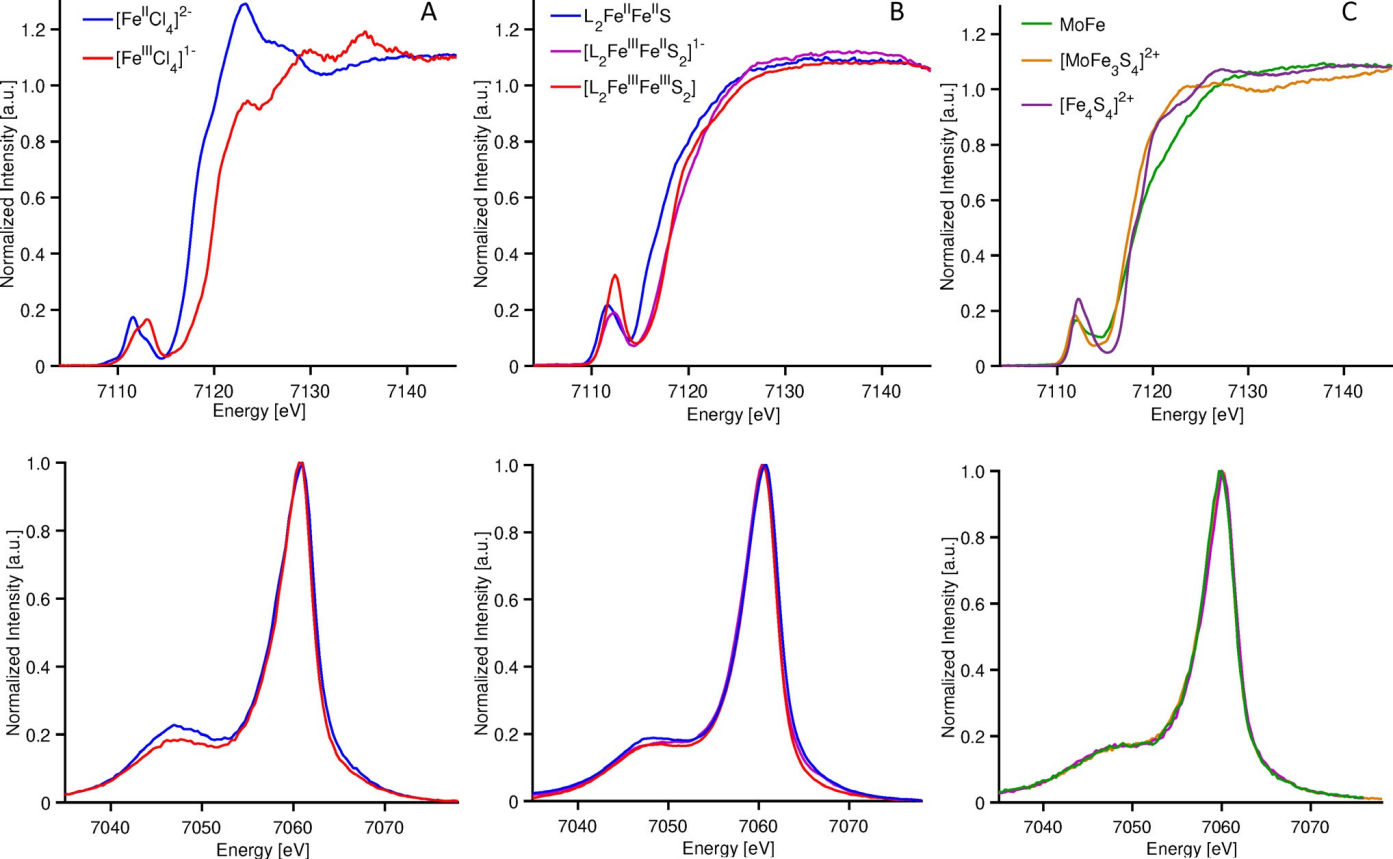
Fe Kβ HERFD‐XAS (top) and Kβ XES (bottom) for A) iron tetrachlorides, B) iron sulfur dimers, and C) the iron sulfur cubanes and MoFe protein of nitrogenase.

In order to discuss the advantages of 1s3p RXES, it is helpful first to illustrate how changes in oxidation state manifest in Fe Kβ HERFD‐XAS and Kβ XES, and how these changes are modulated by covalency. Figure 3 A presents the Fe Kβ HERFD‐XAS data (top) and Kβ XES data (bottom) for [Fe^III^Cl_4_]^1−^ and [Fe^II^Cl_4_]^2−^.

The XAS spectra show the classic changes that one expects to see upon oxidation of the metal center. Namely, the Fe K‐edge shifts up in energy by ∼1.2 eV, reflecting the increase in effective nuclear charge on going from Fe^2+^ to Fe^3+^. In contrast, the changes in the Kβ mainlines are more subtle, with a modest decrease in the intensity of the Kβ′ feature (at ca. 7045 eV) and a slight decrease in the energy of the Kβ_1,3_ maximum (by −0.4 eV) on going from d^6^ *S*=2 Fe^2+^ to d^5^ *S*=5/2 Fe^3+^. In a simple picture, one would expect the splitting between the Kβ_1,3_ and Kβ′ to increase with an increasing number of unpaired d electrons. The fact that the splitting of the Kβ mainline features is somewhat smaller in the *S*=5/2 ferric case than the *S*=2 ferrous case indicates that the increase in covalency (resulting from the Fe−Cl bonds contracting from (2.30±0.01) Å to (2.18±0.01) Å upon oxidation) counters the increase in spin.^[65]^

Figure 3 B shows a comparison of the Fe K‐edge XAS (top) and Kβ XES (bottom) for a series of sulfide bridged iron dimers clusters, identified by their formal oxidation state as diferrous L_2_Fe^II^Fe^II^S, mixed valent [L_2_Fe^II^Fe^III^S_2_]^1−^ and diferric L_2_Fe^III^Fe^III^S_2_. The assignment of oxidation states in this series is based on both structural changes and Mössbauer parameters (Figures S1,2 and Tables S1,2). While the diferrous complex has the expected lowest energy rising edge, the mixed‐valent and diferric complexes are nearly superimposable. Consequently, in this case, the use of the Fe K‐edge as an isolated measure of oxidation state could lead to an incorrect assignment of the physical oxidation state. For the Kβ mainlines, the changes are even smaller. Upon going from the diferrous to the diferric, the Kβ_1,3_ maxima shift by <0.3 eV and the Kβ′ for all three complexes remains constant at 7059.3 eV. This is once again attributed to the canceling effects of covalency and changing d‐count, as has been previously established.[^29^, ^37^, ^61^, ^65^, ^66^] These observations clearly limit the information content of Kβ XES, and also provide a cautionary note against using these data to assess radiation damage.

Both Kβ XES and HERFD‐XAS become less informative as covalency increases. Figure 3 C shows a comparison of the Fe K‐edge XAS (top) and Kβ XES (bottom) of the cubane clusters: [MoFe_3_S_4_]^2+^ (*S*=3/2), [Fe_4_S_4_]^2+^ (*S*=0), and the MoFe protein of nitrogenase. The MoFe protein contains 8 Fe^2+^ ions in its P‐cluster and 4 Fe^2+^/3 Fe^3+^ in its iron–molybdenum cofactor FeMoco,^[67]^ [MoFe_3_S_4_]^2+^ contains 2 Fe^2+^/Fe^3+^ with a delocalized mixed‐valence pair,^[68]^ and [Fe_4_S_4_]^2+^ contains 2 Fe^2+^/2 Fe^3+^. Hence, one would expect the MoFe, containing the highest fraction of Fe^II^ should have the lowest energy edge, however as shown here (Figure 3 C, top) and in previous reports,[^62^, ^69^] this is clearly not the case. Similarly, the corresponding Kβ mainlines also fail to inform on oxidation state changes, with all 3 spectra being effectively superimposable (Figure 3 C, bottom).

Given the ambiguity in both the XAS and XES for the iron sulfur dimer, tetramers and MoFe protein, as well as the small changes in the XES of the iron chlorides, one can reasonably question how useful either method is for assessing oxidation states. Do the data suggest that discussions of specific oxidation state assignments become meaningless in the limit of high covalency, as is sometimes suggested in the literature?[^70^, ^71^] For the iron chloride and iron sulfur series, this is clearly not the case. Both the structural changes and Mössbauer parameters are fully consistent with assigned oxidation states (Figures S1,2 and Tables S1,2). Similarly, combined spectroscopic and computational studies have also clearly established oxidation state differences in the cubanes relative to the all‐ferrous P‐cluster and FeMoco cofactors in the MoFe protein.^[72]^ Hence, the question is, can meaningful and quantifiable differences be obtained from X‐ray spectroscopic approaches in order to assess the electronic structural changes?

### 1s3p RXES of Iron Tetrachlorides

As shown in Figure 1, in a 1s3p RXES experiment, rather than ionizing a 1s electron to the continuum, the electron is resonantly excited to a 3d orbital. This produces a 1s^1^3d^*n*+1^ intermediate state (IS), and a 3p^5^3d^*n*+1^ final state (FS). Thus, the contrasting electronic configurations accessed via resonant (d^*n*+1^) and non‐resonant (d^*n*^) XES will result in distinct multiplet structures, analysis of which could allow for unambiguous assignment of physical oxidation states.

**Figure 4:**
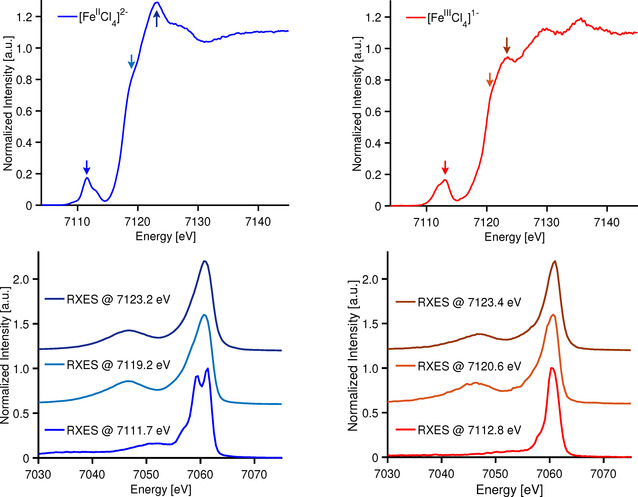
Experimental Fe Kβ HERFD XAS (top) and 1s3p RXES (bottom) on ferrous (blue) and ferric (red) tetrachlorides.

In order to illustrate this hypothesis and show the dependence of RXES on the excitation energy, we present 1s3p RXES of [Fe^II^Cl_4_]^2−^, and [Fe^III^Cl_4_]^1−^, at three different incident excitation energies: one in the pre‐edge and two in the rising edge (Figure 4). Examination of the 1s3p RXES data for [Fe^II^Cl_4_]^2−^, when exciting into the rising edge region at 7119.2 eV and 7123.2 eV shows that the spectra are essentially identical to the Kβ XES, with Kβ_1,3_ maximum at ∼7161 eV and a Kβ′ at ∼7047 eV. This indicates that at the rising edge energies, the 1s electron is being excited into high energy unoccupied orbitals with no impact on the p–d exchange interactions that dominate the Kβ mainline region and there is no added information from the 1s3p RXES. In contrast, the 1s3p RXES spectra of [Fe^II^Cl_4_]^2−^ change dramatically upon resonant excitation into the pre‐edge (1s to 3d). Two clear features appear in the Kβ_1,3_ region with maxima at ∼7059.3 and ∼7061.4 eV, in addition, a weak shoulder is observed at ∼7051.4 eV. The differences in the RXES spectra in the pre‐edge region relative to those in the rising edge clearly demonstrate that a different FS (3p^5^3d^7^ for [Fe^II^Cl_4_]^2−^) has been reached.

Figure 4 (right panel) shows the parallel 1s3p RXES from [Fe^III^Cl_4_]^1−^ with resonant excitations in both the edge and pre‐edge region. Once again, the data obtained with resonant excitation into the edge region are essentially identical to the Kβ XES indicating that, at these energies, the FS is equivalent to the non‐resonant process. Interestingly, for resonant excitation into the pre‐edge region, one observes a Kβ_1,3_ maximum at 7060.6 eV and a weak shoulder at 7057.9 eV. Similar to the ferrous case, upon resonant excitation in the pre‐edge region, no well‐defined Kβ′ feature is observed, distinctly demonstrating that the 1s3p RXES of the ferric species is not simply the equivalent of Kβ XES on a ferrous complex, despite their seemingly equivalent 1s^2^3p^5^3d^6^ FS. This indicates that one must consider the IS that are available to be populated by resonant excitation. In the section that follows, the detailed origins of the differences in Kβ XES and 1s3p RXES are evaluated.

### Non‐resonant versus Resonant XES Multiplets in Iron Tetrachlorides

**Figure 5:**
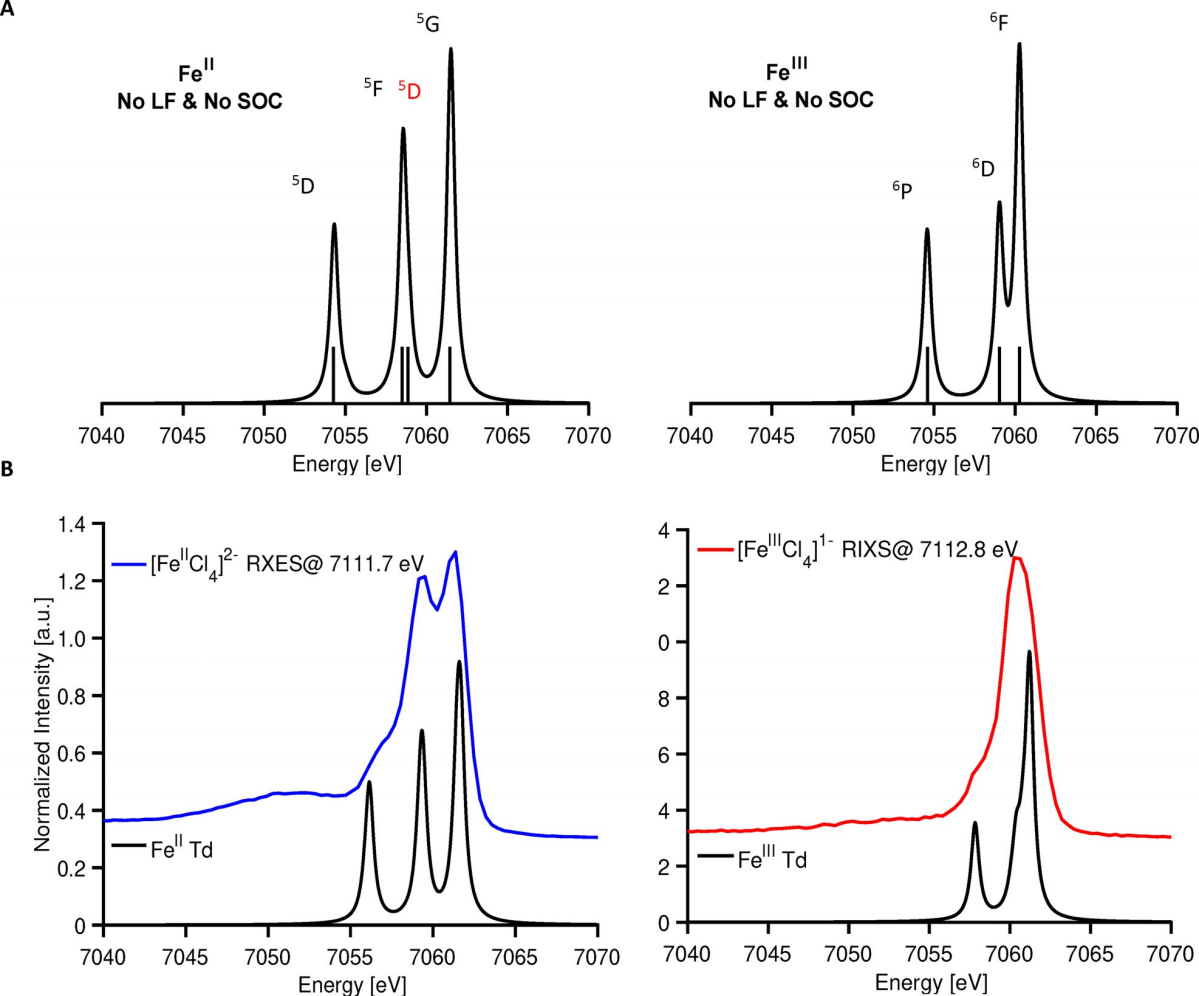
A) Simulated 1s3p RXES spectra for d^6^ (left) and d^7^ (right) and their corresponding multiplets without ligand field inclusion. Multiplets labeled in black are derived from their corresponding d^*n*^ ground state parent term, while those labeled in red are due to an energetically higher parent term. B) Experimental 1s3p RXES on ferrous (left) and ferric (right) tetrachlorides, overlaid with simulated spectra including ligand field and 60 and 45 % SC reduction, respectively.

In order to more rigorously understand the differences in the Kβ XES and 1s3p RXES, it is useful to examine the FS multiplets which are reached in each process. For simplicity, we begin by computing the multiplets involved in the d^6^ and d^5^ Kβ XES spectra (Figure S3). Atomic Russell Saunders terms for these processes are given in Table S4. For the high‐spin d^6^ ferrous case, the ground state term symbol is ^5^D. When a 1s electron is ionized, a 1s^1^3d^6^ state is reached, giving rise to ^4^D and ^6^D configurations when alpha and beta 1s electrons are ionized, respectively. Following the Kβ emission process, a 3p hole is created giving rise to a 3p^5^3d^6^ electron configuration. Coupling the ^2^P configuration of the 3p hole to the ^5^D ground state term symbol gives rise to ^4,6^P, D, and F final state multiplets (Table S4). The multiplet splitting is dominated by 3p–3d exchange, which separates the XES spectrum into a ^4^Γ (Kβ^′^) and ^6^Γ (Kβ_1,3_). In the d^5^ case, a similar picture may be derived, where the FS spectra are instead characterized by splitting of the ^5^Γ and ^7^Γ states. These simple pen and paper predictions are readily captured by atomic multiplet calculations (Figure S3 and Figure S4) and fully consistent with previous assignments for Kβ XES spectra.^[65]^ The 1s3p RXES process at the pre‐edge region, preferentially populates a 1s^1^3d^*n*+1^ IS (Table 1, Figure 5). Following decay via Kβ emission, one arrives at a 1s^2^3p^5^3d^*n*+1^ FS. As there are no *α* 3d holes in either high‐spin Fe^II^ or Fe^III^, only β 1s to 3d transitions are spin allowed. Assuming conservation of spin during the radiative decay process, the β‐decay channel will also dominate. This yields to a Kβ XES spectrum exhibiting ^5^Γ terms for Fe^II^ and ^6^Γ terms for Fe^III^ (Table 1). Hence, upon resonant excitation into the pre‐edge region, the pronounced 3p–3d exchange coupling is no longer the largest contribution to the spectral shape and instead the Kβ′ is effectively absent. This is consistent with the full 1s3p RXES planes (Figure S5)^[73]^ where the pre‐edge region shows intensity only on the Kβ_1,3_ channel.

**Table 1:** Russell–Saunders terms (2S+1L) for the 1s3p RXES process.

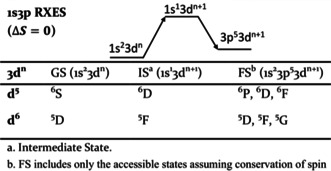

For 1s3p RXES in the pre‐edge region, the ferrous and ferric spectra are dominated by splitting of the FS ^5^Γ and ^6^Γ, respectively. But why is the 1s3p RXES spectrum of the ferrous tetrachloride so strongly structured, while that of the ferric is relatively featureless? Here, our conceptual understanding comes from considering the many‐electron states accessible in the RXES process. In the ferrous case, the 1s3p RXES spectrum comes from a d^7^ configuration for the intermediate and final states. In the valence space, the d^7^ configuration gives rise to a ^4^F term that must be coupled to the ^2^S term for the IS and a ^2^P term for the FS. Therefore, the FS have ^5^D, ^5^F, and ^5^G terms (Table 1). Importantly, there are two ^5^Γ terms that contribute to the spectrum at ∼7059 eV in the ferrous case yielding greater spectral intensity.

This is supported by the multiplet simulation shown in Figure 5 A, which highlights the ^5^D term. This ^5^D term can be seen as deriving from low‐lying ^4^P term of d^7^ metals that is stabilized by configuration interaction. Figure S6 further describes the role of configuration interaction and the identification of ^5^D (^4^P) feature using multiplet simulations.

For the ferric case, the ^5^D term from the d^6^ configuration coupled with ^2^S of the 1s core hole results in a ^6^D IS. In the Kβ XES, ^6^F, ^6^D, and ^6^P FS multiplets may be accessed. In contrast to the ferrous case, there are no low lying multiplets that the ^5^D parent term can interact with, resulting in a more simplified FS multiplet structure relative to that of the ferrous. These trends are validated by multiplet calculations, Figure 5. The 1s3p RXES spectra of both iron tetrachlorides clearly show that resonant excitation into the pre‐edge region allows for the nearly identical Kβ XES spectra to be readily distinguished within the resonant limit. Does this observation continue to hold even for highly covalent iron sulfur clusters?

### Effect of Covalency on 1s3p RXES Spectra

The contribution of covalency to 1s3p RXES spectra was investigated through computational studies in which the Slater Condon parameters (F^2^_pd_ and G^1,3^_pd_) were systematically scaled from 100 % (e.g. the atomic limit) to 40 % (Figure S7). These simulations show that though the spectra are modulated by covalency, the general multiplet structure is maintained even for an unphysical 40 % reduction and the splitting between the ^5^G and ^5^F/^5^D terms is still large enough to be resolved experimentally. To further assess this assumption, data on highly covalent systems are presented in the next section.

### RXES on Highly Covalent Systems: Synthetic Iron Sulfur Dimers Clusters, Iron Sulfur Cubanes and the MoFe Protein of Nitrogenase

**Figure 6:**
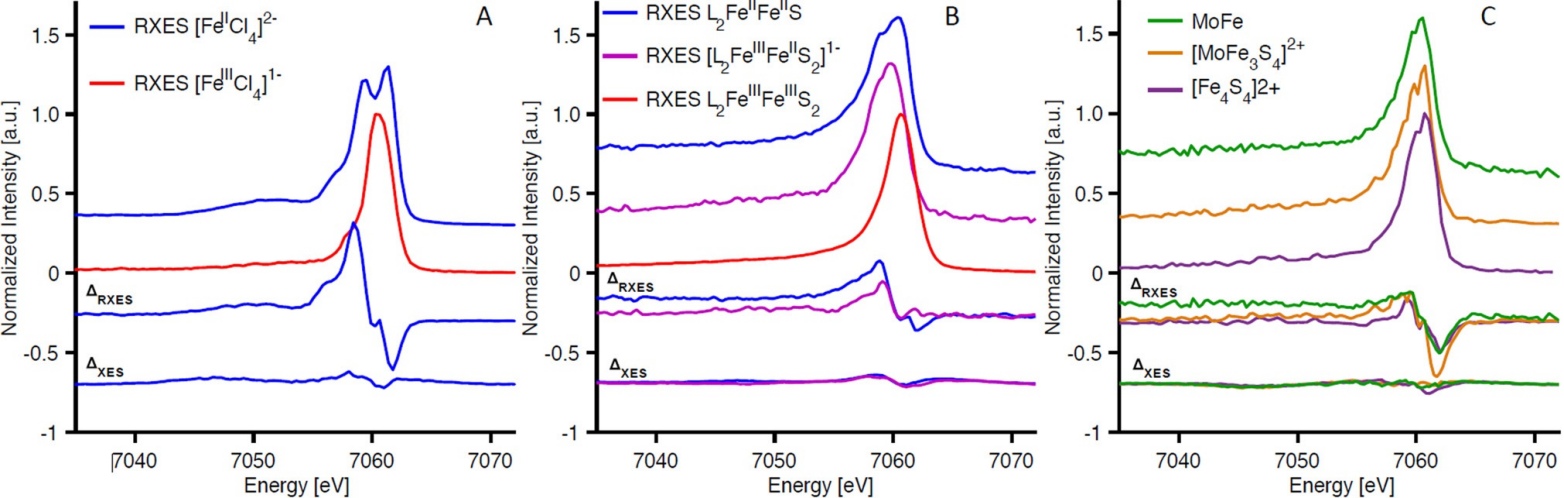
Experimental 1s3p RXES at the pre‐edge, and difference spectra Δ_XES_ and Δ_RXES_ upon subtracting the diferric component for A) iron tetrachlorides, B) iron sulfur dimers, and C) iron tetramers and MoFe protein. Diferric component in subtraction: [Fe^III^Cl_4_]^1−^ (A), and L_2_Fe^III^Fe^III^S (B and C).

The RXES spectra of all molecules are plotted in Figure 6 (positive axis), and difference spectra (Δ_RXES_ and Δ_XES_) generated by subtracting a ferric reference from ferrous species are given following the color of their ferrous parent (negative axis). The all‐ferric reference spectra are chosen to as [Fe^III^Cl_4_]^3−^ for the monomer (Figure 6 A) and L_2_Fe^III^Fe^III^S_2_ for the dimer and tetramers (Figure 6 B and C), to account for the high covalency present in the dimers and tetramers. To evaluate the presence of Fe^II^ in the mixed‐valence clusters and in the MoFe protein versus the 1s3p RXES for Fe^III^, the incident energy chosen for the mixed‐valence samples corresponds to the 1s→3d (Fe^II^). Additional RXES cuts can be found in the Supporting Information (Figure S8 and S9).

Figure 6 A emphasizes the points already made in the preceding section regarding the similarity of the classical XES versus the clear changes in the RXES, now highlighted too by the difference spectra Δ_XES_, and Δ_RXES_. Figure 6 B shows 1s3p RXES at the pre‐edge of the diferrous, mixed valent, and diferric dimers. As was the case for the iron tetrachlorides, resonant excitation clearly distinguishes these three complexes. The advantage of 1s3p RXES over conventional XES is again highlighted by the corresponding difference spectra, showing minor differences for Δ_XES_ in comparison to Δ_RXES_. The changes are more pronounced for the diferrous dimer than the mixed valent dimer. In the diferrous case, resonant excitation into the pre‐edge gives rise to two features (at ∼7058.8 and ∼7060.5 eV), attributed to ^5^Γ derived from the ^4^F and ^4^P parent terms of the d^7^ configuration, as discussed above. In the mixed valent complex, two features are still present, however, the lower energy feature is reduced in intensity, consistent with the fact that only half the irons are in the ferrous form. This is also highlighted by the difference spectrum Δ_RXES_.

Finally, the biomimetic tetranuclear [MoFe_3_S_4_]^2+^ and [Fe_4_S_4_]^2+^ and the MoFe protein of nitrogenase were studied (Figure 6 C). All samples contain Fe^II^, and the resonant excitation into the pre‐edge yields two distinct features split by ∼0.9–1.3 eV at their maxima due to the additional ^5^D term discussed above. Once again, inspection of the Δ_RXES_ and Δ_XES_ highlights the ability of 1s3p RXES at the pre‐edge region to identify the presence of ferrous iron, which is not possible using the Kβ XES spectra alone. Future studies including 1s3p RXES full planes collected with higher resolution instruments may allow for a more rigorous quantification of the changes in electronic structure, enabling better distinction between the different intermediate states. Nevertheless, these results demonstrate that even in highly covalent systems, meaningful differences in 3d metal valency persist in the 1s3p RXES data.

## Conclusion

The work presented here illustrates the ability of 1s3p RXES to identify physical oxidation states which may be hidden in standard Kβ XES and K‐edge XAS experiments. This is achieved by resonantly exciting into the pre‐edge region of the XAS spectrum, giving rise to a FS with a d^*n*+1^ configuration. By comparing the multiplet structure of Kβ XES to that of 1s3p RXES, both the ground state and final state multiplets may be experimentally accessed, allowing for the physical oxidation state to be assigned in a far more robust manner than is possible in standard XAS or XES experiments.

RXES and XES are complementary in the assessment of the oxidation state. However, there are certain advantages of RXES. For all d^*n*^ configuration with *n*≥5, only β excitations are allowed in the pre‐edge region, resulting in a RXES spectrum in which only the states of maximum multiplicity contribute. Combined with the higher achievable resolutions in 1s3p RXES relative to Kβ XES, this allows for a more quantitative assessment of the electronic structure. Furthermore, the multiplet structure in 1s3p RXES persists upon strong covalent modulation, unlike in the non‐resonant Kβ XES case.

The results presented should be readily translatable to all high‐spin first‐row TMs of any d^*n*^ count. The splitting of the 1s3p RXES Kβ_1,3_ feature for Fe^II^ results from the ^5^F term that is reached in its IS (s^1^d^7^) and the existence of a low‐lying excited state with the same spin multiplicity with which mixing can occur via configuration interaction. This same phenomenon should occur for all TMs that reach an F term in the IS. Based on these observations, general groupings can be made for all first‐row TMs with high‐spin configurations:

- A splitting of the 1s3p RXES Kβ_1,3_ feature will be observed for all TMs in which an F term is its IS. E.g. d^1^, d^2^, d^6^ and d^7^ ground state configurations.
- For d^*n*≥5^ TMs the 1s3p RXES at the pre‐edge, will be absent from the Kβ′ feature.
- A splitting of the Kβ_1,3_ feature in Kβ XES should be observed for TMs with F ground state terms, providing experimental resolution is sufficient (e.g. d^2^, d^3^, d^7,^ and d^8^ configurations).

The general pairings of resonant and non‐resonant XES multiplet behavior thus provide a means to distinguish electronic structural ambiguities for a range of 3d^*n*^ counts.

In recent years, the literature has seen increasing applications of Kβ XES to a wide range of TM systems, including in situ and operando studies of catalysts,[^74^, ^75^, ^76^, ^77^] transformations of materials under high pressure,[^78^, ^79^] and time‐resolved (TR) studies of enzymatic systems.[^80^, ^81^] However, in many of these cases the information conveyed by Kβ XES is ambiguous and their conclusions are at times controversial. This study makes it clear that 1s3p RXES provides an opportunity to further the chemical information content of XES even on biological systems. The inclusion of 1s3p RXES to the MoFe protein of nitrogenase in the present work shows not only the feasibility of the experiment on enzymatic systems (data collection time was 8 minutes/incident energy), but also highlights that RXES could significantly increase the information about the electronic structure obtained in TR studies.

In our view, this work brings us one step closer to realizing the vision of Linus Pauling who said, “If scientific progress continues, the next generation may have a theory of valency that is sufficiently precise and powerful to permit chemistry to be classed along with physics as an exact science.” Although we are not there yet, we have added another tool to the chemists’ toolbox to bring the community one step closer to realizing this goal.

## Conflict of interest

The authors declare no conflict of interest.

## Supporting information

As a service to our authors and readers, this journal provides supporting information supplied by the authors. Such materials are peer reviewed and may be re‐organized for online delivery, but are not copy‐edited or typeset. Technical support issues arising from supporting information (other than missing files) should be addressed to the authors.

Supplementary
