# Supplementary material for: Probing Physical Oxidation State by Resonant X‐ray Emission Spectroscopy: Applications to Iron Model Complexes and Nitrogenase
Source: Angew Chem Int Ed Engl. 2021 Mar 23;60(18):10112–21. doi: 10.1002/anie.202015669 (PMC8252016; doi:10.1002/anie.202015669)
Supplement: Supplementary file 1 — Supplementary [file ANIE-60-10112-s001.pdf]

Angewandte Chemie
Eine Zeitschrift der Gesellschaft Deutscher Chemiker

Supporting Information

Probing Physical Oxidation State by Resonant X-ray Emission Spectroscopy: Applications to Iron Model Complexes and Nitrogenase

*Rebeca G. Castillo, Anselm W. Hahn, Benjamin E. Van Kuiken, Justin T. Henthorn, Jeremy McGale, and Serena DeBeer**

anie_202015669_sm_miscellaneous_information.pdf

SUPPORTING INFORMATION

Table of Contents

EXPERIMENTAL METHODS 3

GENERAL CONSIDERATIONS
SAMPLE PREPARATION (FIGURES S1 & S2)
DATA COLLECTION
MULTIPLET SIMULATION

FIGURE S3 8

TABLE S4 & FIGURE S4 9

FIGURE S5 10

IDENTIFYING PARENT TERMS 11

FIGURE S6

FIGURE S7 12

FIGURE S8 13

FIGURE S9 14

REFERENCES 15

Experimental Methods

General Considerations

Unless indicated otherwise, all manipulations were performed using oven-dried glassware in an M-Braun nitrogen-atmosphere glovebox or on a Schlenk line using standard Schlenk techniques. Molecular sieves were activated by heating to 200°C for 48 hours under high vacuum. Tetrahydrofuran and diethyl ether were purchased anhydrous from Sigma, further dried over sodium/benzophenone ketyl, vacuum transferred before use, and stored over 4Å molecular sieves. Naphthalene was purchased from Sigma and purified via vacuum sublimation. Potassium graphite and $SSbPh_3$ were purchased from Sigma and used as received.

Mössbauer Spectroscopy. Zero-field Mössbauer spectra were recorded with a ^{57}Co source in a Rh matrix using an alternating constant-acceleration Mössbauer spectrometer operated in transmission with a liquid helium cryostat (Oxford Instruments) for measurements at 80 K. The γ-source was kept at room temperature. All isomer shifts are quoted relative to iron metal at 300 K. Mössbauer spectra were fit with Lorentzian doublets using the program *mf* developed by E. Bill at MPI CEC. All parameters are collected in **Table S1**.

X-ray Crystallography. The crystal structures of compounds $\mathbf{L_2Fe^{III}Fe^{III}S_2}$ and $\mathbf{[K(THF)_6][L_2Fe^{III}Fe^{II}S_2]{\bullet}2THF}$ were determined using a Bruker-Nonius Kappa Mach3/APEX II diffractometer equipped with a Mo IμS anode and INCOATEC Helios mirror optics (λ = 0.71073 Å). Diffraction data was collected at 100 K in a nitrogen cryo-stream. Final cell constants were obtained from least squares fits of several thousand strong reflections. Intensities of redundant reflections were used to correct for absorption using the SADABS program.[2] The structure was readily solved by Patterson methods and subsequent difference Fourier techniques. The Siemens ShelXTL software package[3] was used for solution of the structures, and ShelXL-2013[4] was used for structure refinement. All nonhydrogen atoms were anisotropically refined and hydrogen atoms bound to carbon were placed at calculated positions and refined as riding atoms with isotropic displacement parameters. Figures of the crystal structures in the manuscript were generated using the Olex2 software[5] and ORTEP plots presented in the supplementary information were generated using the ShelXTL software package. CCDC 1920935 and 2022318 contain the supplementary crystallographic data for this paper. These data can be obtained free of charge from The Cambridge Crystallographic Data Centre via www.ccdc.cam.ac.uk/data_request/cif. Selected bond metrics are presented in **Table S2**. Crystallographic refinement details are collected in **Table S3**.

Sample preparation: $[NEt_4][Fe^{III}Cl_4]$ was purchased from Sigma-Aldrich and used as received. $[NEt_4]_2[Fe^{II}Cl_4]$ and $L_2Fe^{II}Fe^{II}S$ (L^{1-} = 2,6-diisopropylphenyl *β*-diketiminate ligand, see Figure 2) were synthesized according to reported procedures.[6] Both tetranuclear clusters $[Fe_4S_4Cl_4]^{2-}$ ($\mathbf{[Fe_4S_4]^{2+}}$) and $(Et_4N)[(Tp)MoFe_3S_4Cl_3]$[17] ($\mathbf{[MoFe_3S_4]^{2+}}$) were synthesized as previously reported.[7-8] The MoFe protein (300uM protein, buffer: 50mM Tris, 200mM NaCl, 5mM MgCl2) from A. Vinellandi nitrogenase was prepared following published protocols.[9]

Synthesis of $L_2Fe^{III}Fe^{III}S_2$. A solution of $SSbPh_3$ (0.0998 g, 0.256 mmol) in Et_2O (3 mL) was added to a solution of $L_2Fe^{II}Fe^{II}S$ (0.2445 g, 0.250 mmol) in Et_2O (3 mL) in a 20 mL vial and the mixture stirred overnight. The resulting dark purple suspension was filtered through a glass frit and the collected purple-maroon precipitate washed with additional Et_2O (10 mL). The resulting maroon solid was then dried under vacuum to yield 0.1544 g (59%) of crude diferric complex $L_2Fe^{III}Fe^{III}S_2$. By Mössbauer, the isolated product contains a small (5-10% over 3 preparations) unidentified ferrous impurity that could not be removed by further purification attempts due to the insolubility of the product. Crystals suitable for X-ray diffraction could be prepared by layering a solution of $SSbPh_3$ (10 mg) in Et_2O (2 mL) onto a solution of L_2Fe_2S (25 mg) in Et_2O (2 mL) and letting the mixture stand at room temperature overnight.

Synthesis of $[K(THF)_6][L_2Fe^{III}Fe^{II}S_2]{\bullet}2THF$. A solution of $KC_{10}H_8$ was generated by adding KC_8 (0.0237 g, 0.175 mmol) to a solution of $C_{10}H_8$ (0.0262 g, 0.204 mmol) in THF (3 mL). After stirring for 30 minutes, the resultant dark green $KC_{10}H_8$ mixture was added to a stirred suspension of the maroon $L_2Fe_2S_2$ (0.1554 g, 0.154 mmol) in THF (3 mL). The mixture was stirred for 1 hour, at which point the maroon precipitate had been consumed to afford a dark red-brown mixture. The mixture was filtered through a glass microfiber filter pad to remove graphite, rinsing with additional THF (ca. 3 mL). The dark red filtrate was then concentrated under vacuum. The residue was triturated with Et_2O (3 mL) and filtered on a glass microfiber filter pad. The

resulting dark red solid was washed with additional Et_2O until the filtrate ran clear (ca. 4 mL). The dark red solid was then resolubilized in THF (ca. 6 mL), filtered through the glass microfiber pad, further concentrated to half-volume under vacuum, and stored at –35 °C to afford 0.0624 g (25% yield) of dark red crystals (suitable for XRD). UV-vis (THF, 21 °C), λ_{max} (nm), ε ($M^{-1}cm^{-1}$): 380, $1.48x10^4$; 425, $9.4x10^3$; 550, $6.0x10^3$; 610, $3.7x10^3$; 715, $1.7x10^3$.

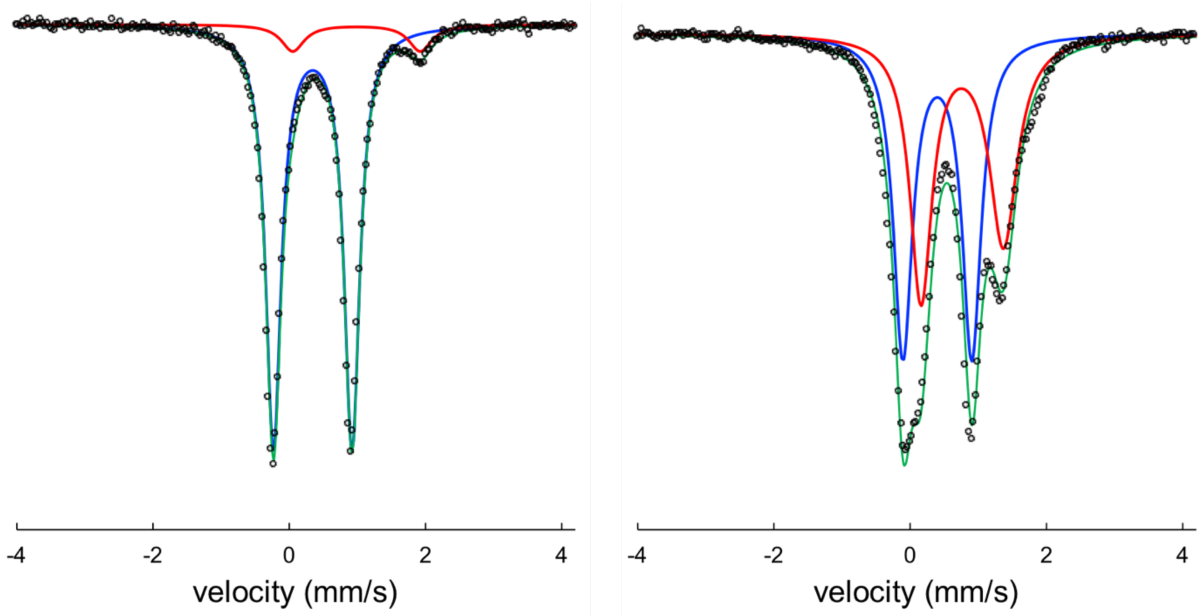

Figure S1. Zero field Mössbauer spectra of **$L_2Fe^{III}Fe^{III}S_2$** (left) and **$[L_2Fe^{III}Fe^{II}S_2]^{1-}$** (right) recorded at 80K. The data are shown as open circles and the Lorentzian fittings for individual quadrupole doublets as red and blue lines, with the sum of the fittings shown as green lines. The spectrum for **$L_2Fe^{III}Fe^{III}S_2$** is fitted with an 8% unidentified ferrous impurity (red line). Fitting parameters are presented in Table S1.

Table S1. Mössbauer fit parameters for complexes **$[Fe^{III}Cl_4]^{1-}$**, **$[Fe^{II}Cl_4]^{2-}$**, **$L_2Fe^{III}Fe^{III}S_2$**, **$[L_2Fe^{III}Fe^{II}S_2]^{1-}$**, and **$L_2Fe^{II}Fe^{II}S$**

Compound	Formal ox. State	δ (mm/s)	$\|\Delta_{EQ}\|$ (mm/s)	fwhm[a] (mm/s)	η[b] (a.u.)	relative intensity (%)
**$[Fe^{III}Cl_4]^{1-}$**	Fe^{3+}	0.32	0.11	0.39	1	100
**$[Fe^{II}Cl_4]^{2-}$**	Fe^{2+}	1.03	2.54	0.33	1	100
**$L_2Fe^{III}Fe^{III}S_2$**	$Fe^{3+}Fe^{3+}$	0.34	1.15	0.28	1	92
	Fe^{2+}	0.99[c]	1.87[c]	0.41[c]	1[c]	8[c]
**$[L_2Fe^{III}Fe^{II}S_2]^{1-}$**	Fe^{3+}	0.40	1.01	0.34	1	51
	Fe^{2+}	0.76	1.21	0.39	1.27	49
**$L_2Fe^{II}Fe^{II}S$**	$Fe^{2+}Fe^{2+}$	0.86[c]	0.58[c]	--	--	100[c]

[a]Full width at half-maximum. [b]Asymmetry parameter. [c]Unidentified ferrous impurity. [d]Data taken from reference 5.

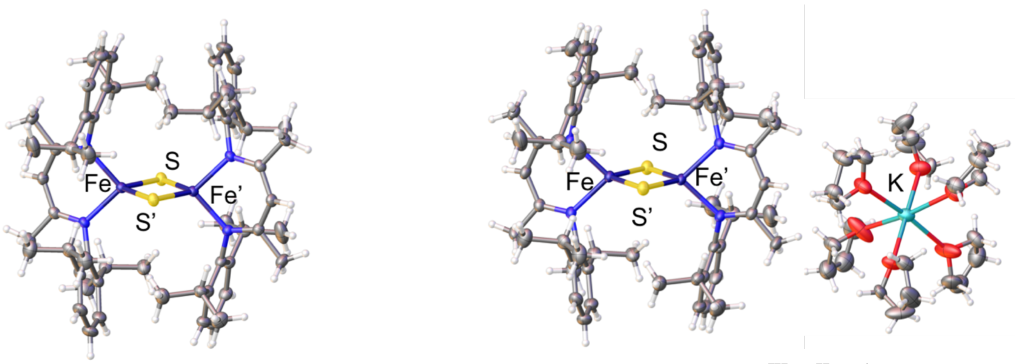

$L_2Fe^{III}Fe^{III}S_2$ $[L_2Fe^{III}Fe^{II}S_2]^{1-}$

Figure S2. Single crystal X-ray diffraction structures of $\mathbf{L_2Fe^{III}Fe^{III}S_2}$ (left) and $\mathbf{[K(THF)_6][L_2Fe^{III}Fe^{II}S_2]{\bullet}2THF}$ (right) shown with 50% probability ellipsoids. Solvent molecules of crystallization in $\mathbf{[K(THF)_6][L_2Fe^{III}Fe^{II}S_2]{\bullet}2THF}$ are omitted for clarity. Nitrogen atoms are shown in blue, oxygen atoms in red, carbon atoms in gray, and hydrogen atoms in white. Selected bond metrics are reported in Table S1.

Table S2. Selected bond metrics for complexes $\mathbf{L_2Fe^{III}Fe^{III}S_2}$, $\mathbf{[L_2Fe^{III}Fe^{II}S_2]^{1-}}$, and $\mathbf{L_2Fe^{II}Fe^{II}S}$

	$L_2Fe^{III}Fe^{III}S_2$	$[L_2Fe^{III}Fe^{II}S_2]^{1-}$	$L_2Fe^{II}Fe^{II}S$
Fe–Fe (Å)	2.816	2.807	3.564[a]
$Fe–S_{avg}$ (Å)	2.210	2.238	2.298[a]
$Fe–N_{avg}$ (Å)	2.028	2.098	2.035[a]
$\angle Fe–S–Fe_{avg}$ (°)	79.2	77.7	101.7[a]
$\angle N–Fe–N_{avg}$ (°)	91.6	88.0	93.1[a]
$\angle S–Fe–S_{avg}$ (°)	79.2	77.7	--

[a] Data taken from reference [6].

Table S3. Crystallographic refinement details for complexes $\mathbf{L_2Fe^{III}Fe^{III}S_2}$ and $\mathbf{[K(THF)_6][L_2Fe^{III}Fe^{II}S_2]•2THF}$.

Compound	$\mathbf{L_2Fe^{III}Fe^{III}S_2}$	$\mathbf{[K(THF)_6][L_2Fe^{III}Fe^{II}S_2]•2THF}$
CCDC	2022318	1920935
empirical formula	$C_{58}H_{82}Fe_2N_4S_2$	$C_{90}H_{146}Fe_2KN_4O_8S_2$
formula wt	1011.09	1627.02
T (K)	100	100
a, Å	22.308(4)	18.569(1)
b, Å	14.647(3)	19.177(2)
c, Å	16.268(3)	25.835(3)
alpha, deg	90	90
beta, deg	90.074(3)	95.249(9)
gamma, deg	90	90
V, $Å^3$	5316(2)	9161(2)
Z	4	4
crystal system	Monoclinic	Monoclinic
space group	C2/c	C2/c
d_{calc}, g/cm^3	1.263	1.18
θ range, deg	2.781-30.000	2.649-29.999
μ, mm^{-1}	0.655	0.461
abs cor	Gaussian	Gaussian
GOF	1.081	1.075
R_1,[a] wR_2[b] (I > 2sig(I))	0.0569, 0.1477	0.0604, 0.1557

[a] $R_1 = \sum\left||F_o| - |F_c|\right| / \sum|F_o|$. [b] $wR_2 = [\sum[w(F_o^2 - F_c^2)^2] / \sum[w(F_o^2)^2]]^{1/2}$

***Data Collection**:* All X-ray spectroscopic data were measured at either beamline ID26, at the European Synchrotron Radiation Facility (ESRF) or the GALAXIES beamline, at SOLEIL synchrotron. The ESRF storage ring was operating at 6 GeV, and the experiment was done performing at 7/8 +1 filling mode with 200 mA current. SOLEIL storage ring operates at 2.75 GeV, and the measurements were performed in 4/4 top-up filling mode with 500 mA current. A double crystal monochromator Si(111) was utilized for selection of the incident beam energy. The incident fluxes at both beamlines were $>10^{13}$ photons/s at ID26 and $>10^{12}$ photons/s at GALAXIES beamline. The beam size at the sample was 0.1 mm (v) x 1 mm (h) and 0.08 mm (v) x 1.5 mm (h) for ID26 and Galaxies beamlines, respectively. The incident energy was calibrated by setting the first inflection point of a Fe foil to 7111.2 eV. X-ray emission from the sample was measured using a Johann-type spectrometer equipped with an array of four (GALAXIES) or five (ID26) spherically bent Ge (620) analyzer crystals aligned in the Rowland geometry in combination with a silicon drift diode detector. To avoid attenuation of the fluorescence emission, a helium-filled bag was placed filling the flight path between sample, analyzer crystals, and detector. The spectrometers have an averaged measured resolution, including the monochromator's contribution, of ~1.0 and ~1.6 eV at the ESRF and Soleil beamlines, respectively. All data were measured at the ESRF, except the iron sulfur dimer data. Due to the resolution of the monochromator, it is important to be aware that resonant excitation can result in a distribution of core-excited states rather than a single state, and thus, the final states resulting in the RXES spectra have some contribution of all the states that are being populated. Full analysis of each excitation state within the pre-edge region could be obtained by measuring high resolution 1s3p RXES planes using high resolution incident beam, however, it is beyond the scope of the present work.

To minimize sample damage, the incident beam was attenuated when necessary by placing aluminium filters before the sample. The energy of the XES spectrometer was calibrated by setting the Kβ XES maxima (Kβ′ and $K\beta_{1,3}$ features) of Fe_2O_3 to 7045.2 eV and 7060.6 eV, respectively. All spectra were normalized to the incident flux by using a photodiode detector. Samples were maintained at temperatures of 10-40K by using a LHe flow cryostat (ID26) or a He cryostream (GALAXIES).

For both experiments, radiation damage assessments were carried for each individual compound. Dwell time per spot for each compound was assessed by collecting consecutive short energy range XAS scans of 10 sec/scan. Only scans showing no evidence of radiation damage were included in the final averages.

$K\beta_{1,3}$-detected XAS was collected in an energy range of 7105-7170 eV in 0.2 eV steps and longer energy scans were taken for normalization purposes, with an energy range of 7100-7800 eV. The long energy scans were averaged together, pre- and post-edge background subtracted, and the edge jump normalized to one.

Non-resonant and resonant Kβ XES measurements were collected in an energy range of 7030-7070 eV in 0.25 eV steps. The incident energies for the 1s3p RXES measurements were selected from the $K\beta_{1,3}$-detected XAS measurements. XES data was normalized to the maximum of the emission Intensity.

Multiplet simulation. Non-resonant Kβ XES and 1s3p RXES simulations were performed in the framework of atomic multiplet theory. Three atomic shells are included in the calculations: 1s, 3p and 3d. The many-body Hamiltonians include the effects of electron-electron interactions and the ligand field effects. The two-electron interactions are parameterized by the typical Slater-Condon factors, and the ligand field is considered in cubic symmetry. In previous studies[10-11] it was noted that the pd exchange integrals $G^{1,3}_{pd}$ largely dominate the spectral shape of XES in transition metals, and the F^2_{pd} integrals make a minor contribution. Thus, the effects of the Slater-Condon factors F^2_{pd} and $G^{1,3}_{pd}$ on the calculated spectra were studied by scaling the values from 40 % to 80% (default value), where a scaling factor of 45-60% was found to reproduce better the iron tetrachlorides experimental results due to the high covalency of the samples. It is noted that spin–orbit interaction is neglected. This has the effect that $\hat{S}^2$ still commutes with the Hamiltonian, and the unambiguous spin state assignments can be made. The Hamiltonian is constructed and diagonalized for the initial, intermediate, and final states necessary for describing the Kβ XES and 1s3p RXES processes. This is achieved by restricting the number of electrons in each atomic shell to a specified configuration. For the RXES simulations, the configurations for initial, intermediate and final states are $1s^23p^63d^n$, $1s^13p^63d^{n+1}$, and $1s^23p^53d^{n+1}$, respectively. For non-resonant XES calculations, the initial and final states possess the $1s^13p^63d^n$ and $1s^23p^53d^n$ configurations, respectively. Spectra were constructed using the Kramers-Heisenberg equation for RXES and the standard expression for the emission rate for XES.[12] In the case of RXES simulations, all states contributing to the ground state term were included as equally weighted initial states for the RXES processes. For XES, the core-ionized states were determined by the sudden approximation applied to the ground state wave function.[13] All simulations were carried out using the EDRIXS set of python modules extended locally

with tools for computing XES spectra.[14] To facilitate comparison of the calculations with experimental RXES spectra, the calculated RXES were average over a ~0.5 eV window of incident energies and the incident energy direction of the 1s3p calculated planes have a broadening of 0.5 eV. Thus, the calculated 1s3p RXES approaches a total of 1 eV spectral broadening. This allows us to simulate a range of intermediate states that may be populated in the RXES process. Furthermore, simulated spectra were plotted with 1.5 eV and 0.3 eV Lorentzian broadening for non-resonant XES and RXES, respectively.

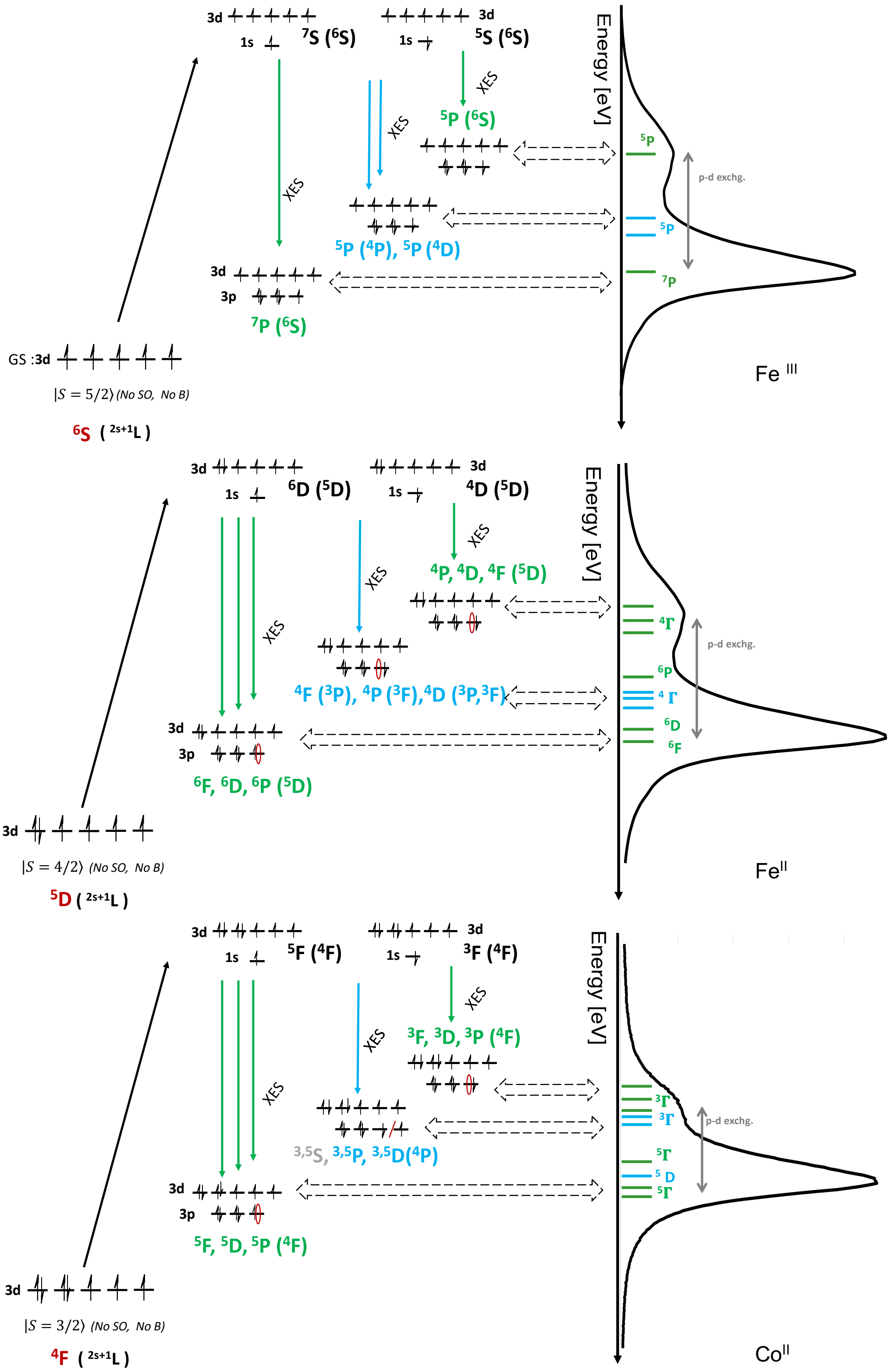

Figure S3. Scheme describing Kβ Non-Resonant XES processes for d^5, d^6, and d^7.

Table S4. Russel Sanders Terms (2S+1L) for the Kβ XES process

Kβ XES

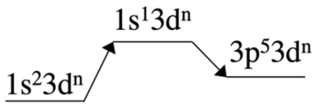

$3d^n$	GS ($1s^23d^n$)	IS* ($1s^13d^n$)	FS ($1s^23p^53d^n$)
**d^5**	^6S	$^{5,7}S$	$^{5,7}P$
**d^6**	^5D	$^{4,6}D$	$^{4,6}P$, $^{4,6}D$, $^{4,6}F$
**d^7**	^4F	$^{3,5}F$	$^{3,5}D$, $^{3,5}F$, $^{3,5}G$

*Note IS is referring to Ionized State (1s e- ionization process)

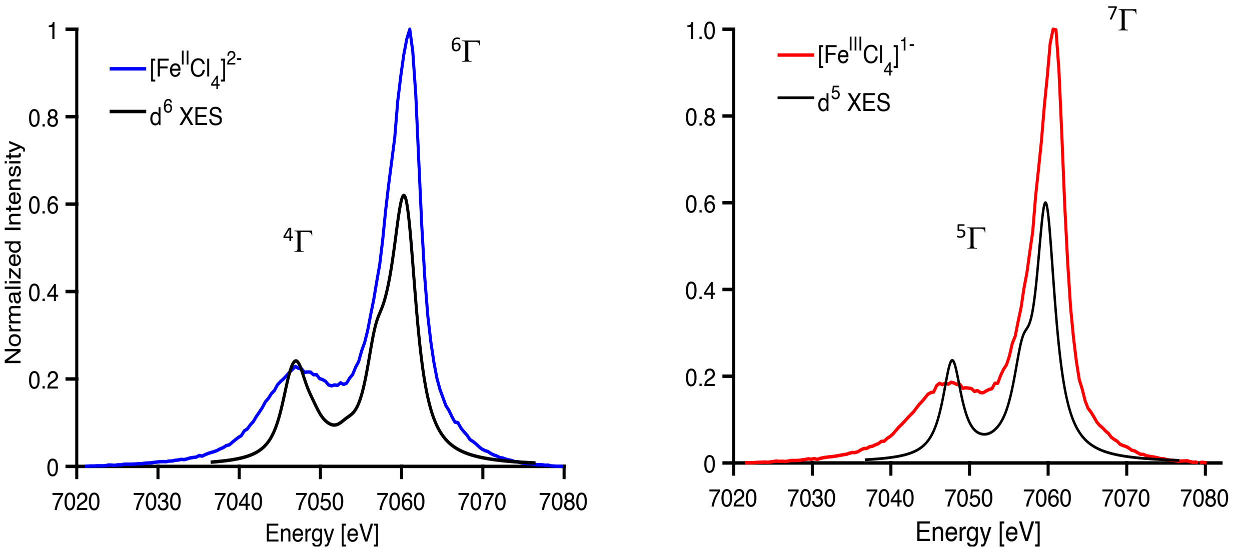

Figure S4. Experimental Kβ XES on ferrous (blue) and ferric (red) tetrachlorides and simulated (shell) Kβ XES for d^6 and d^5 (black) using 1.5 eV Lorentzian broadening and 60% and 45 % SC reduction.

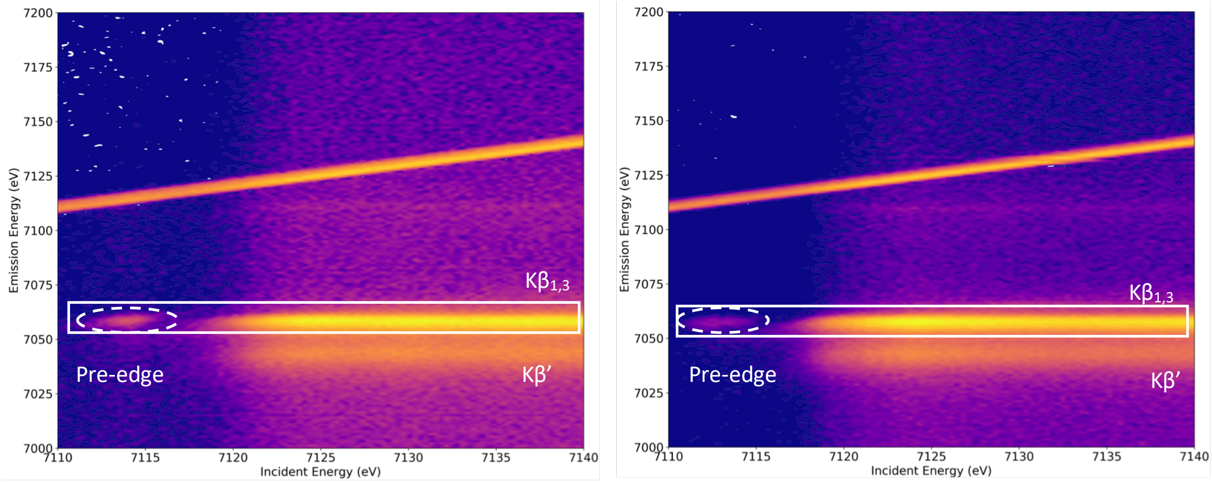

Figure S5. Normalized 1s3p RXES plane for $[Fe^{III}Cl_4]^{1-}$ (left) and $[Fe^{II}Cl_4]^{2-}$(right)[1] measured at SuperXAS-SLS using a von Hamos spectrometer, as reported in the thesis of Anselm W. Hahn [1]. The pre-edge region is found to align with the $K\beta_{1,3}$ feature, while no pre-edge intensity is seen at the energy of the Kβ’ feature. This is consistent with the pre-edge region of the RXES plane being dominated only by 1s to 3d transitions on the β-channel. It is important to note these planes were collected with a different experimental setup than the one used in the present work, and differences in both energy calibration and experimental resolution preclude more detailed correlations with the present data.

Identifying parent term for Fe^{II}

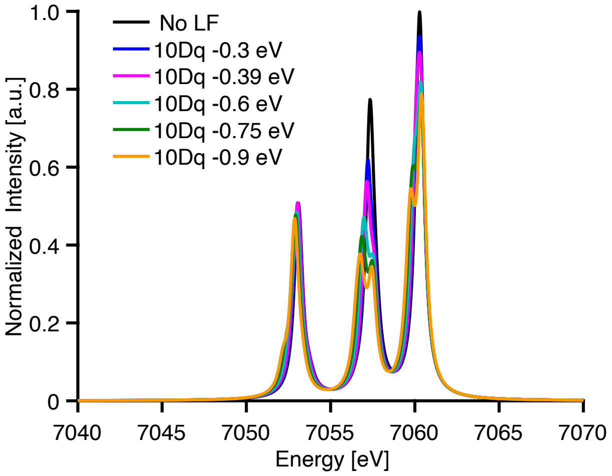

Figure S6. Simulated RXES Fe^{II} td scaling 10Dq.

The ability of the d^7 ground state term (^{4}F) to have symmetry allowed mixing with the low lying ^{4}P state upon application of even a weak ligand field is what gives rise to the intrinsically different spectral shape in the K$\beta_{1,3}$ spectral region. Thus, in order to assign term symbols to the spectral features arising in the multiplet simulation, the ligand field (LF) parameters were varied. By scaling the 10Dq value in the d^6 simulation for $[Fe^{II}Cl_4]^{2-}$ up to 300%, we can identify the presence of the ^{5}D (^{4}P) in the spectra as it is the most sensitive feature to changes in the LF. **Figure S5** shows that the intensity of the second highest energy peak (~7058 eV) is the most affected by changing in the ligand field and consequently it holds the ^{5}D (^{4}P) term.

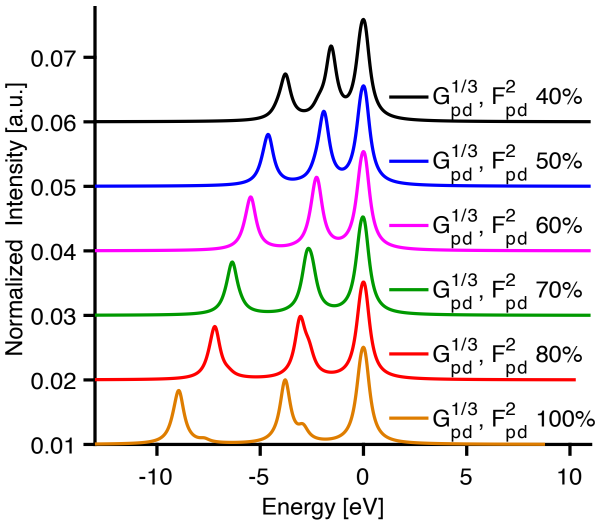

Figure S7. Simulated d^6 1s3p RXES varying the scaling factor of $G^{1/3}_{pd}$ and F^2_{pd} SC parameters. For better comparison between simulations, all spectra have been aligned to the maximum of the highest intensity peak. Simulated spectra with 0.3 eV Lorentzian broadening .

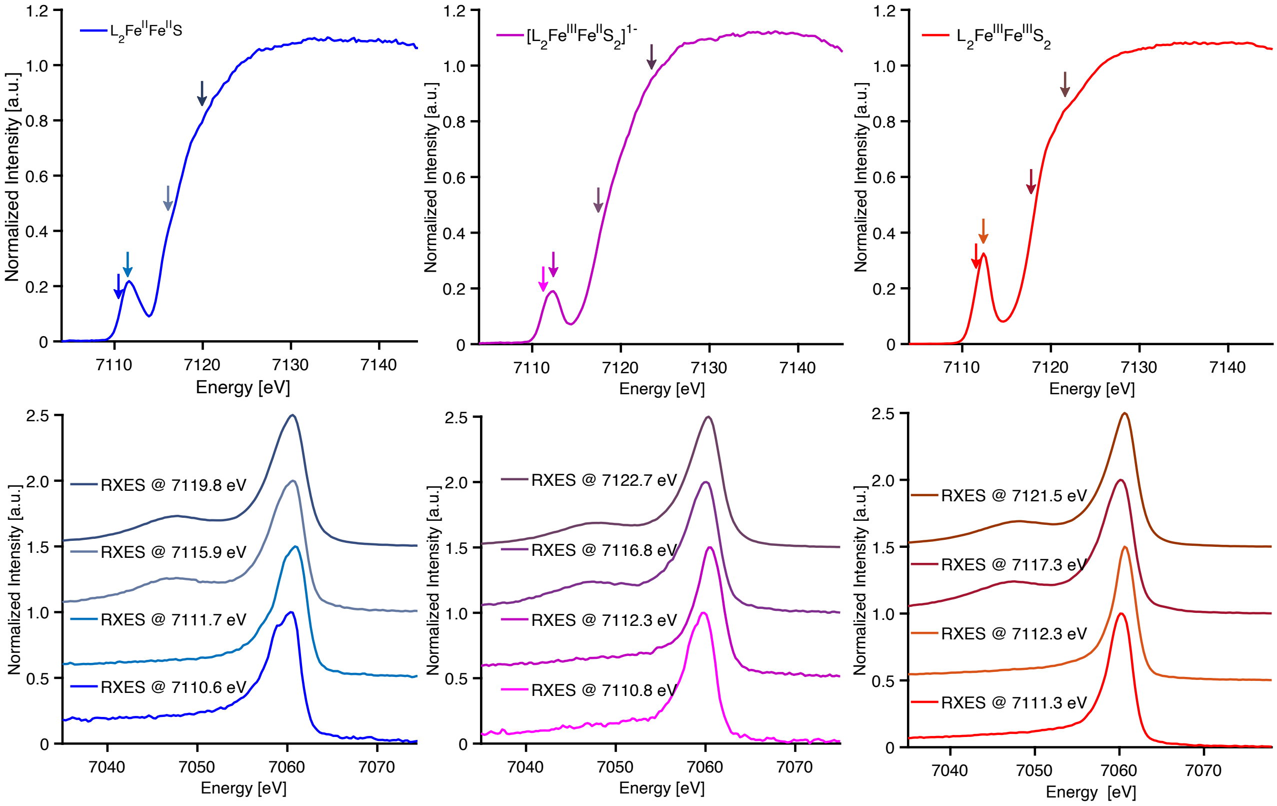

Figure S8. Experimental Kβ HERFD XAS (Top) and 1s3p RXES (bottom) of ferrous (blue), mixed valence (purple) and ferric(red) iron-sulfur dimers.

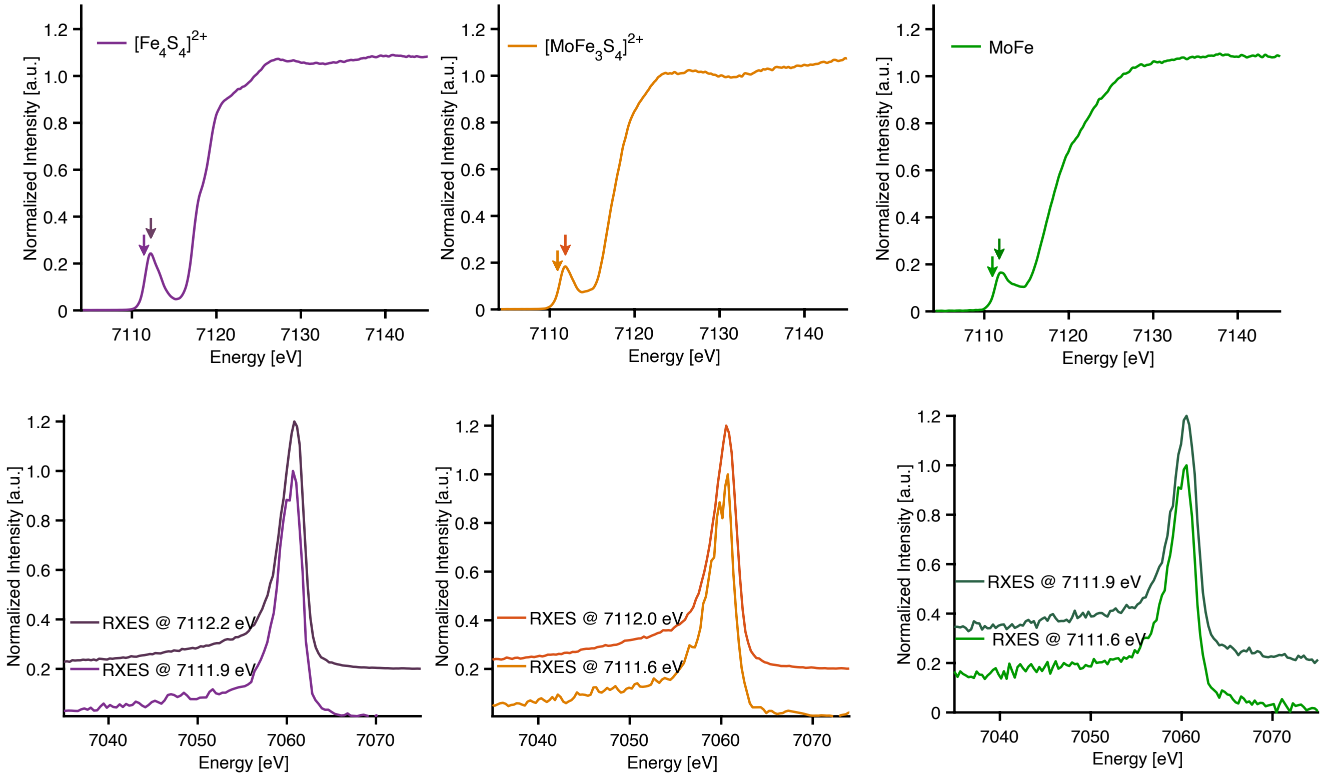

Figure S9. Experimental Kβ HERFD XAS (Top) and 1s3p Kβ RXES (bottom) of and the iron sulfur cubanes and MoFe protein of nitrogenase.

References

[1] A. W. Hahn, Ruhr-Universität Bochum **2018**.
[2] in *SADABS-2014/5 - Bruker AXS area detector scaling and absorption correction, Madison, WI, USA.*
[3] in *ShelXTL, 6.14, Madison, WI, USA, 2003*.
[4] G. M. Sheldrick, G. M. ShelXL Version 2013/2, *Madison, WI, USA, 2013*.
[5] O. V. Dolomanov, L. J. Bourhis, R. J. Gildea, J. A. K. Howard, H. Puschmann, *J. Appl. Crystallogr.* **2009**, *42*, 339-341.
[6] J. Vela, S. Stoian, C. J. Flaschenriem, E. Münck, P. L. Holland, *J. Am. Chem. Soc.* **2004**, *126*, 4522-4523.
[7] D. V. Fomitchev, C. C. McLauchlan, R. H. Holm, *Inorg. Chem.* **2002**, *41*, 958-966.
[8] G. B. Wong, M. A. Bobrik, R. H. Holm, *Inorg. Chem.* **1978**, *17*, 578-584.
[9] T. Spatzal, M. Aksoyoglu, L. Zhang, S. L. A. Andrade, E. Schleicher, S. Weber, D. C. Rees, O. Einsle, *Science* **2011**, *334*, 940.
[10] R. G. Castillo, J. T. Henthorn, J. McGale, D. Maganas, S. DeBeer, *Angew. Chem. Int. Ed.*, *59*, 12965-12975.
[11] C. J. Pollock, M. U. Delgado-Jaime, M. Atanasov, F. Neese, S. DeBeer, *J. Am. Chem. Soc.* **2014**, *136*, 9453-9463.
[12] N. Lee, T. Petrenko, U. Bergmann, F. Neese, S. DeBeer, *J. Am. Chem. Soc.* **2010**, *132*, 9715-9727.
[13] P. S. Bagus, E. S. Ilton, C. J. Nelin, *Surf. Sci. Rep.* **2013**, *68*, 273-304.
[14] Y. L. Wang, G. Fabbris, M. P. M. Dean, G. Kotliar, *Comput. Phys. Commun.* **2019**, *243*, 151-165.
